# Effect of periosteal resection on longitudinal bone growth in a mouse model of achondroplasia

**DOI:** 10.1016/j.bonr.2020.100708

**Published:** 2020-08-13

**Authors:** Shinya Kaneko, Masaki Matsushita, Kenichi Mishima, Yasuhiko Takegami, Shiro Imagama, Hiroshi Kitoh

**Affiliations:** a Department of Orthopaedic Surgery, Nagoya University Graduate School of Medicine, Nagoya, Japan; b Department of Orthopaedic Surgery, Aichi Children's Medical and Health Center, Obu, Japan

**Keywords:** Achondroplasia, Fibroblast growth factor receptor 3, Mouse, Periosteal resection

## Abstract

Achondroplasia (ACH) is the most common form of short-limbed skeletal dysplasia. Patients with ACH sometimes undergo lower limb lengthening to get functional and psychological achievements. The periosteal resection (PR) is a known mechanism to increase longitudinal bone growth without osteotomy, although the results are not predictable. It could be alternative for limb lengthening in a minimally invasive technique. The purpose of this study is to evaluate the effect of PR on acceleration of bone growth in a mouse model of ACH (*Fgfr3*^ach^). We performed a circumferential resection of periosteum on the proximal tibia to both wild-type and *Fgfr3*^ach^ mice at the age of four weeks. The second PR was done one week later in each mouse, which was subsequently sacrificed at the age of six weeks for micro-computed tomography (micro-CT) scan and histological examinations. We measured tibial bone length, bone volume, and metaphyseal trabecular bone parameters, including bone volume/tissue volume (BV/TV), trabecular thickness (Tb.Th), trabecular number (Tb.N) by reconstructed micro-CT images. We also quantified the entire width of the growth plate of the proximal tibial from the sections stained with hematoxylin and eosin. Tibial bone length and bone volume of the PR side were significantly larger than the sham side in wild-type mice, while they were not statistically significant in *Fgfr3*^ach^ mice. The BV/TV and Tb.N in the metaphysis were significantly decreased in the PR side of both mice. The histological analysis revealed that the growth plate of the proximal tibia was significantly wider in the PR side of wild-type mice while it showed no difference in width between the PR side and the sham side in *Fgfr3*^ach^ mice. PR promoted longitudinal bone growth in wild-type mice, but it exhibited only a marginal effect on bone growth in *Fgfr3*^ach^ mice.

## Highlights

• Periosteal resection (PR) enhances longitudinal bone growth in wild-type mice.
• PR increases width of the growth plate but decreases metaphyseal trabecular.
• The effect of PR on bone growth is less in a mouse model of achondroplasia.

## 1 Introduction

Achondroplasia (ACH) is a common skeletal dysplasia with shorten-limbed short stature caused by gain-of-function mutation in fibroblast growth factor receptor 3 (FGFR3) gene (Horton et al., 2007). Limb lengthening surgery is a treatment option for short stature in ACH. Patients with ACH who have redundant soft tissues can hopefully gain approximately 20 cm of height after tibial and femoral lengthenings (Donaldson et al., 2015). Extensive limb lengthening, however, needs a long treatment period and decreases patients' quality of life due to commonly associated complications during the treatment such as infection, joint contracture, and fracture (Kitoh et al., 2004). Minimally invasive treatments are desired to gain the height in ACH children.

The periosteal stripping (Jenkins et al., 1975) and periosteal division (Dimitriou et al., 1988) have been reported as a less invasive mean to enhance the longitudinal bone growth clinically and experimentally. Experimentally, Halanski et al. (2016) indicated that the periosteal partial transection or resection achieved more promoting bone growth compared to the periosteal transection of the full length of the tibia in a rabbit model. They also showed that repeated periosteal transection led to better bone growth than a single procedure. Finally, they found that growth acceleration was occurred in the growth plate of the same bone with periosteal procedure. Clinically, the combination procedure of periosteal stripping and periosteal division achieved limb length equalization in eight of 11 patients who had 3–13 cm of limb length discrepancy (LLD) after approximately two years treatment (Limpaphayom and Prasongchin, 2011). The periosteal stripping and division increased bone length by 2.0 ± 1.6% compared with contralateral non-operated side in ten children with ACH (Edwards et al., 1994). These clinical case series, however, did not demonstrate a predictable growth rate after the treatment.

The purpose of this study is to evaluate the growth promotion effect of periosteal procedure in a mouse model of ACH (*Fgfr3*^ach^) and to explore the feasibility of this procedure as an alternative treatment for the limb lengthening surgery in ACH children.

## 2 Materials and methods

### 2.1 Mice

We employed wild-type mice and *Fgfr3*^ach^ mice (FVB background) expressing an activated *FGFR3*, containing the G380R mutation responsible for ACH in the growth plate using the Col2a1 promoter and enhancer sequences (Naski et al., 1998). The *Fgfr3*^ach^ mice were kindly provided by Dr. David M. Ornitz at Washington University. The mice were housed under 12 h light-dark cycle and were provided with water and standard commercial diet freely. All experiments were carried out in accordance with protocols approved by the Animal Care and Use Committee of our institute.

### 2.2 Periosteal resection surgery

**Fig. 1:**
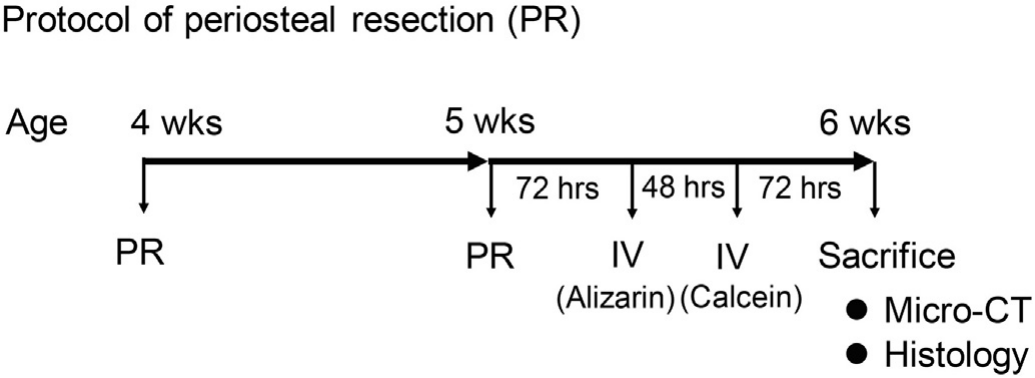
Protocol of periosteal resection (PR) surgery. The PR surgery was performed at the age of four and five weeks. The animals were sacrificed at the age of six weeks and subjected to micro-computed tomography (micro-CT) scan and histology. The intravenous (IV) administration of alizarin and calcein were performed 120 and 72 h before the sacrifice, respectively.

Under inhalation anesthesia by using 2.0% Isoflurane, an anterior longitudinal incision was made on the left lower leg by the length of proximal half tibia with a scalpel using microscope. After anterior tibial muscle was gently retracted from the tibial periosteum, the 10 mm wide of the periosteum was circumferentially removed just below the patellar tendon from the proximal tibia by using scrape and tweezer. Sham operation was performed in the contralateral right side with an incision of skin and fascia. In this way, we performed periosteal resection (PR) to four-week-old mice, and the second PR was subsequently performed one week later (Fig. 1). Since normal periosteum was not found at the second PR, we just removed the scar tissue circumferentially.

### 2.3 Radiographic analysis

The mice were sacrificed at the age of six weeks, and subjected to micro-computed tomography (micro-CT) scan (0.5 mm Al filter, voxel size 0.9 μm, 50 kV, 500 μA; SkyScan1176, Bruker) for evaluating bone length, bone volume, and anterior bowing angle of the tibia. After the reconstruction using the Skyscan NRecon software, the images were analyzed by three-dimensional (3D) algorithms in Skyscan CTAn software according to the manufacture's instructions. The bone length was determined by measuring between proximal and distal central articular surfaces by employing coronal planes of reconstructed 2D images. The tibial bone volume was also measured from the reconstructed 3D images. The anterior bowing angle was defined as the angle between proximal and distal bone axes of the tibia upper the tibiofibular junction by using sagittal plane of reconstructed 2D image (Murray et al., 1996). We further evaluated the trabecular bone of the proximal tibia. The section from 215 μm to 1940 μm below the growth plate was selected as metaphyseal trabecular region of interest (ROI). The bone volume/tissue volume (BV/TV), trabecular thickness (Tb.Th), and trabecular number (Tb.N) were calculated as indices of metaphyseal trabecular compartments.

### 2.4 Histological analysis

The tibia was fixed in 4% paraformaldehyde at room temperature for 24 h. After washing with phosphate buffered saline, the samples were decalcified in 10% EDTA solution at 4 °C for three weeks and then embedded in paraffin. Coronal thin sections (3 μm) were cut and stained with hematoxylin and eosin. The sections were stained with type X collagen (Col X) antibody (Abcam) for immunohistochemistry. The Images were captured with a BZ-X710 (Keyence, Osaka, Japan) microscope. The measurements were made at the median quarter, center, and lateral quarter across the width of each growth plate. We averaged these measurements as a longitudinal length of growth plate. The growth plate area and hypertrophic zone area of the proximal tibial growth plate were quantified by using Image J software based on 200 times magnified images.

Additionally, alizarin complexone (0.015 mg/g IV) and calcein (0.005 mg/g IV) were used for pulsed fluorochrome labeling. Alizarin and calcein were administered intravenously 120 and 72 h before the sacrifice, respectively (Fig. 1). The tibiae were frozen in cooled hexane and embedded with super cryoembedding medium (SCEM) according to Kawamoto's film method (Kawamoto, 2003). We sliced the tibiae into 10 μm thick section of coronal plane using a tungsten carbide blade, and evaluated by a BZ-X710 (Keyence, Osaka, Japan) fluorescence microscope. The longitudinal length of the calcein-stained area and unstained area were measured by using images captured with the microscope to evaluate the bone growth rate in the proximal tibial growth plate. We chose three points for the measurement of each sample, including median quarter, center, and lateral quarter. These measurements were averaged to give a final growth rate. Alizarin and calcein were optimally viewed with a 510- to 560-nm and a 470-nm excitation filter, and 590-nm and 525-nm barrier filter, respectively. The metaphyseal areas of no signal and green signal indicated the bone growth area during 72 h and 48 h, respectively. These areas were quantified by Image J software.

### 2.5 Statistical analysis

Data were expressed as mean ± SD with relative ratio compared with sham-operated side. Group means were compared using Student's *t*-test. The *p* values less than 0.05 were considered significant. All analyses were performed using EZR (Saitama Medical Center, Jichi Medical University, Saitama, Japan) (Kanda, 2013).

## 3 Results

### 3.1 Periosteal resection increased bone growth

**Fig. 2:**
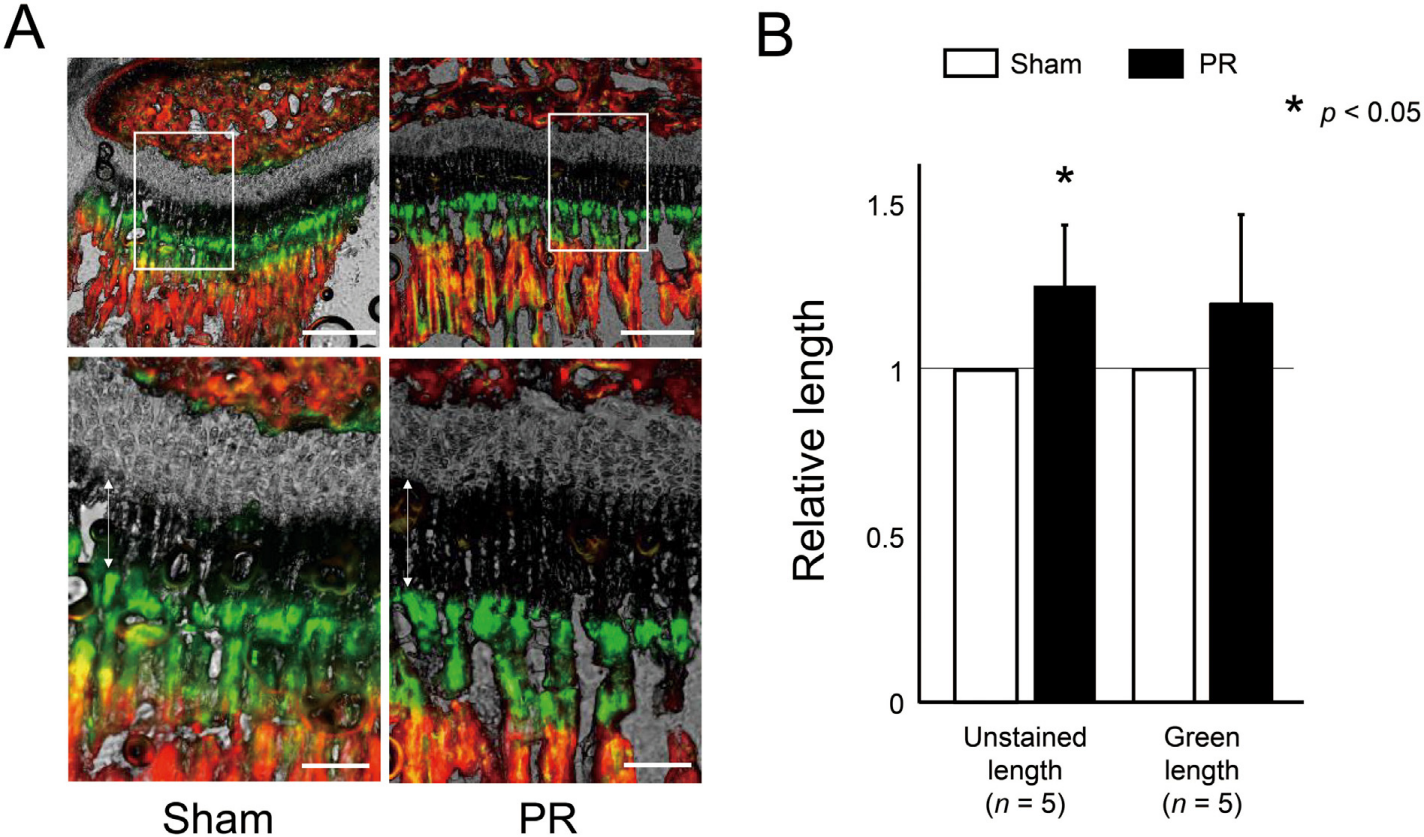
Endochondral ossification was promoted after PR. (A) The represented sections show the metaphysis labelled with alizarin and calcein of wild-type mice. Green and red areas were stained with calcein and alizarin, respectively. The unstained area and green area represent the area grown by 72 and 48 h, respectively. Squared parts are magnified in each lower image. Double arrows indicate the unstained area in the metaphyseal trabecular. Scale bares indicate 300 μm in upper images and 100 μm in lower images. (B) Mean and SD of relative longitudinal length of the unstained area and green area in wild-type mice. The longitudinal length of the unstained area was significantly increased after PR. The statistical differences shown on each graph are analyzed by paired *t*-test. (For interpretation of the references to color in this figure legend, the reader is referred to the web version of this article.)

**Supplementary Fig. S1:**
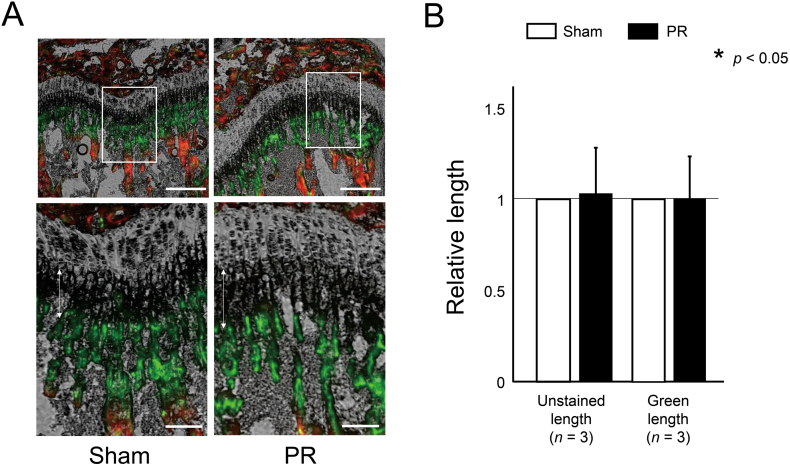
The effect of PR on endochondral ossification in *Fgfr3*^ach^ mice was not found in the fluorochrome labeling sections. (A) The represented sections show the metaphysis labelled with alizarin and calcein of *Fgfr3*^ach^ mice. Green and red areas were stained with calcein and alizarin, respectively. The unstained area and green area represent the area grown by 72 and 48 h, respectively. Squared parts are magnified in each lower image. Double arrows indicate the unstained area in the metaphyseal trabecular. Scale bares indicate 300 μm in upper images and 100 μm in lower images. (B) Mean and SD of relative longitudinal length of the unstained area and green area in *Fgfr3*^ach^ mice. The longitudinal length of both areas was not increased after PR. The statistical differences shown on each graph are analyzed by paired *t*-test.

According to the current protocol (Fig. 1), we initially performed PR to wild-type mice to confirm the effect of PR on promoting bone growth, and demonstrated that longitudinal bone growth in metaphyseal trabecular was apparently promoted in the fluorochrome labeling sections (Fig. 2A). We quantified the bone growth after PR by measuring the longitudinal length of the unstained area and green area in metaphyseal trabecular. The PR increased the longitudinal length of the unstained and the green area (Fig. 2B). On the other hand, PR did not increase both areas in *Fgfr3*^ach^ mice (Supplementary Fig. S1A and B).

### 3.2 Periosteal resection increased bone length and bone volume

**Fig. 3:**
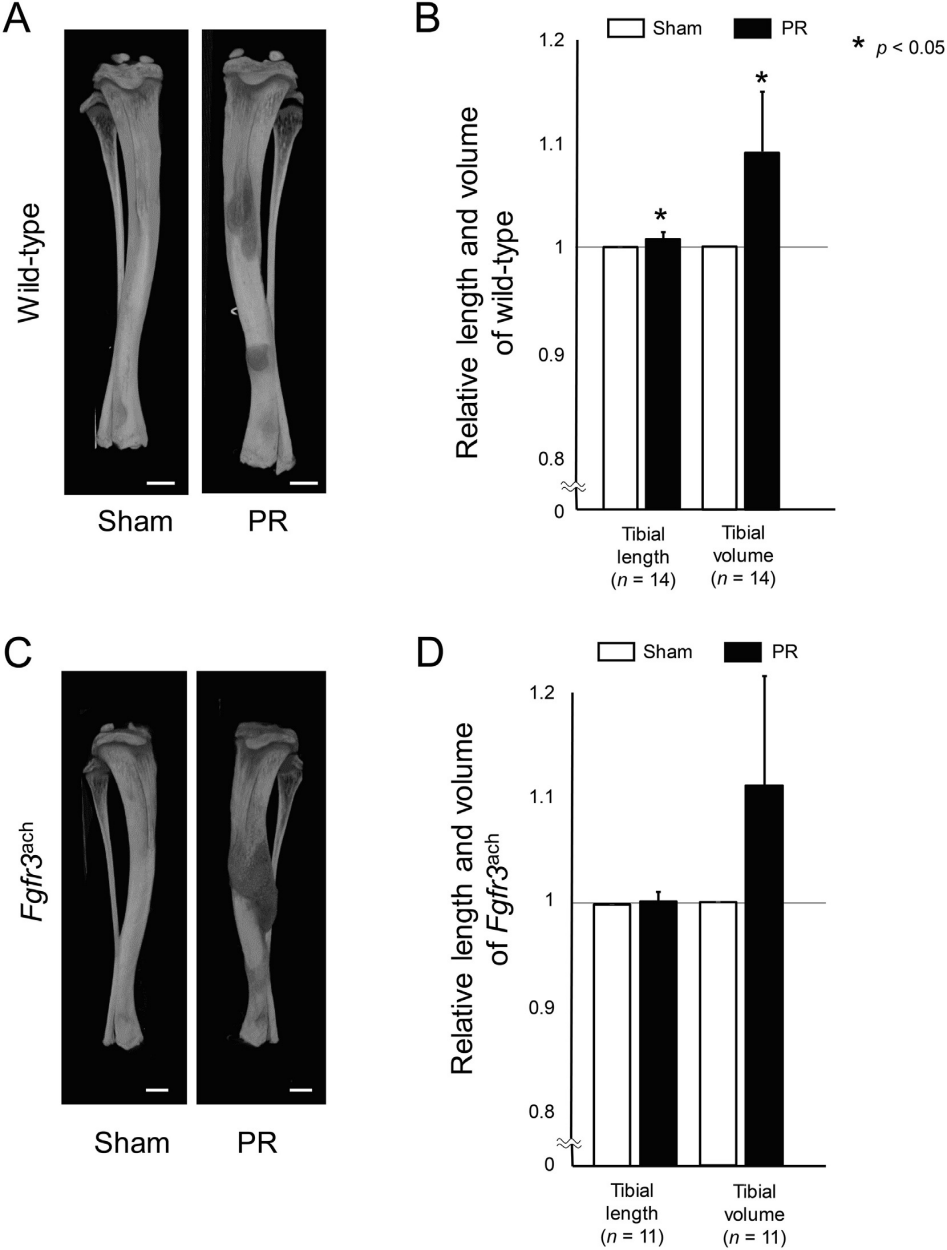
Tibial bone length and volume were increased after PR. (A, C) Representative three-dimensional (3D) images reconstructed from micro-CT scan show the tibiae after PR and sham surgery (Sham) in wild-type (A) and *Fgfr3*^ach^ mice (C). Scale bares indicate 500 μm. (B, D) Mean and SD of each relative length and volume reconstructed 3D image in wild-type (B) and *Fgfr3*^ach^ mice (D) are indicated. The bone length and volume were significantly increased after PR compared with those in Sham in wild-type mice. Similarly in *Fgfr3*^ach^ mice, these values were increased after PR. The statistical differences shown on each graph are analyzed by paired *t*-test.

Next, we evaluated the effect of PR on the bone length and bone volume. Tibia in wild-type mice after PR was significantly larger than that of the sham side (Fig. 3A). The averaged bone length and bone volume of tibia were significantly increased after PR by 0.71 ± 0.72% and 8.87 ± 5.64% compared to the sham side (Fig. 3B). On the other hand, the PR increased the bone length and volume of the tibia, which did not reach statistically significant in *Fgfr3*^ach^ mice (Fig. 3C and D).

**Supplementary Fig. S2:**
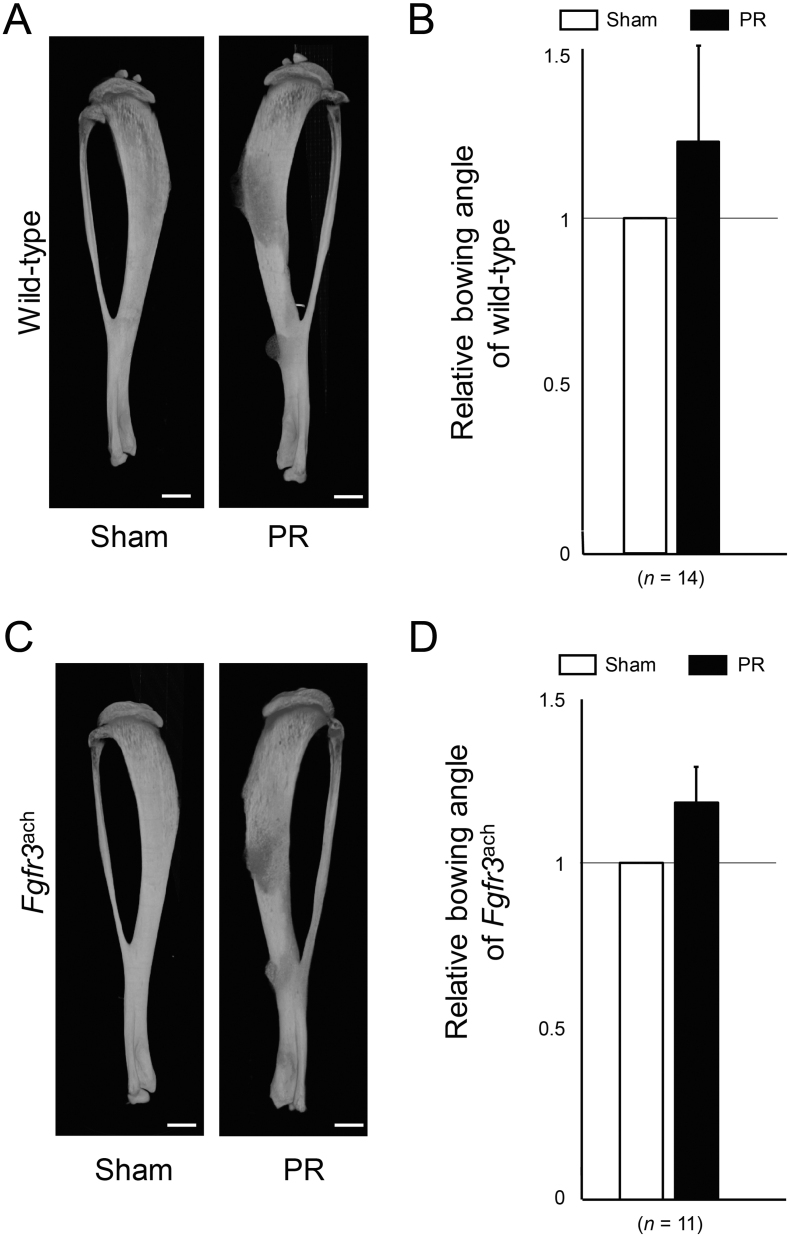
Anterior bowing was increased by PR. (A, C) Representative 3D images reconstructed from micro-CT scan show the tibiae with PR and Sham in wild-type (A) and *Fgfr3*^ach^ mice (C). Scale bares indicate 500 μm. (B, D) Mean and SD of each relative bowing angle of wild-type (B) and *Fgfr3*^ach^ mice (D) are indicated. The bowing angle was increased in PR of both mice.

We further evaluated the bone alignment from reconstructed micro-CT scan images. Advanced bowing of the tibia was apparent after PR (Supplementary Fig. S2A). Relative anterior bowing angle was increased after PR in wild-type mice (Supplementary Fig. S2B). Similar results were obtained after PR in *Fgfr3*^ach^ mice (Supplementary Fig. S2C and D).

### 3.3 Periosteal resection decreased metaphyseal trabecular

**Fig. 4:**
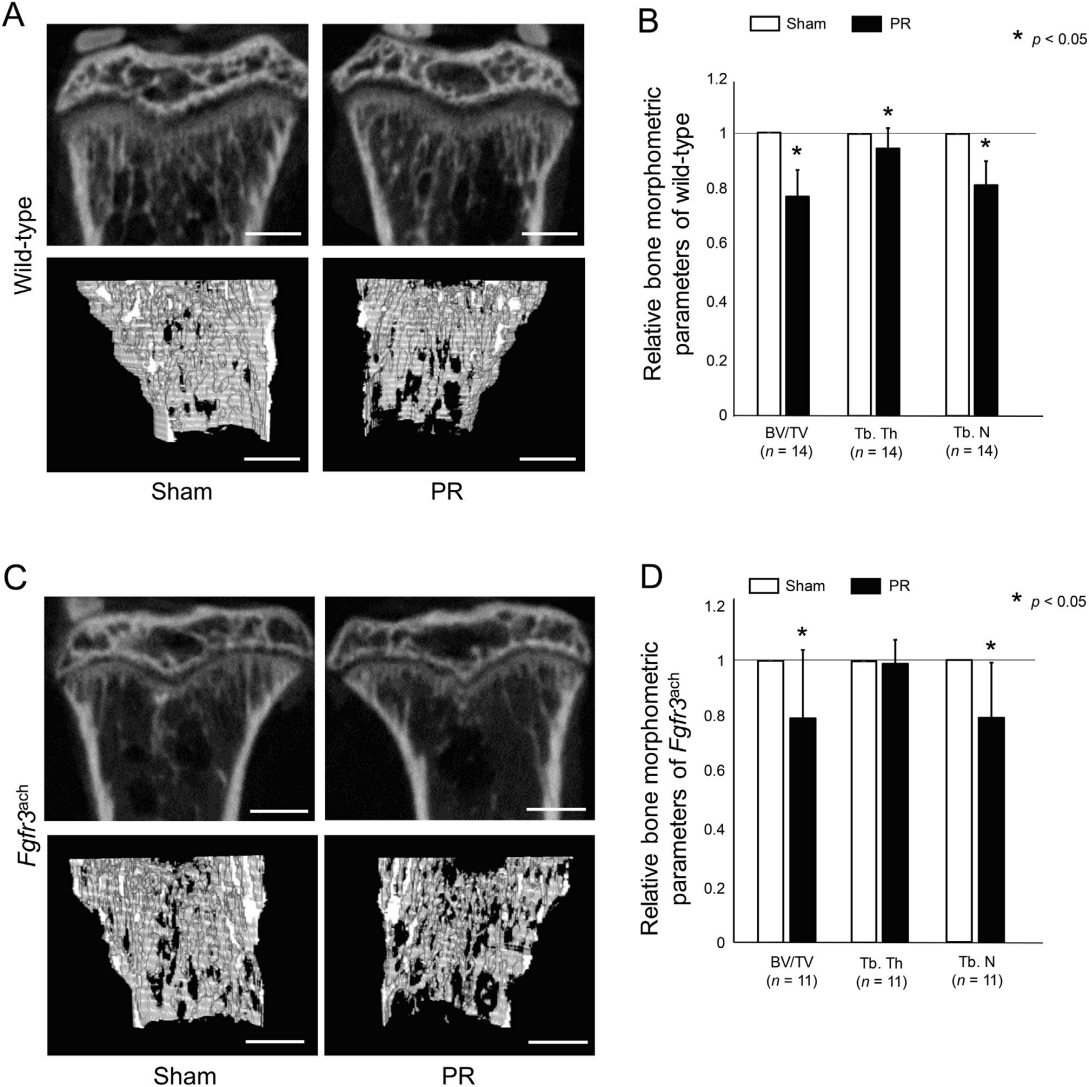
Trabecular bone was decreased after PR. (A, C) Representative reconstructed micro-CT images of the proximal tibial revealed that metaphyseal bone mineral density was decreased after PR in wild-type (A) and in *Fgfr3*^ach^ mice (C). Upper panels show 2D images of the proximal tibia and lower panels show 3D images of the trabecular bone architecture. Scale bares indicate 300 μm. (B, D) Mean and SD of each relative parameter of bone morphometry are indicated. The bone volume/total volume (BV/TV), trabecular thickness (Tb.Th), and trabecular number (Tb.N) of wild-type mice were significantly decreased after PR compared with those in Sham. Similarly in *Fgfr3*^ach^ mice, these parameters were decreased after PR. The statistical differences shown on each graph are analyzed by paired *t*-test.

We next quantified the effect of PR on metaphyseal trabecular. The trabecular bone was decreased after PR in both wild-type and *Fgfr3*^ach^ mice (Fig. 4A and C). The BV/TV, Tb.Th, and Tb.N were significantly decreased after PR in wild-type mice (Fig. 4B). The PR also significantly decreased BV/TV and Tb.N in *Fgfr3*^ach^ mice (Fig. 4D).

### 3.4 Periosteal resection increased the width of the growth plate

**Fig. 5:**
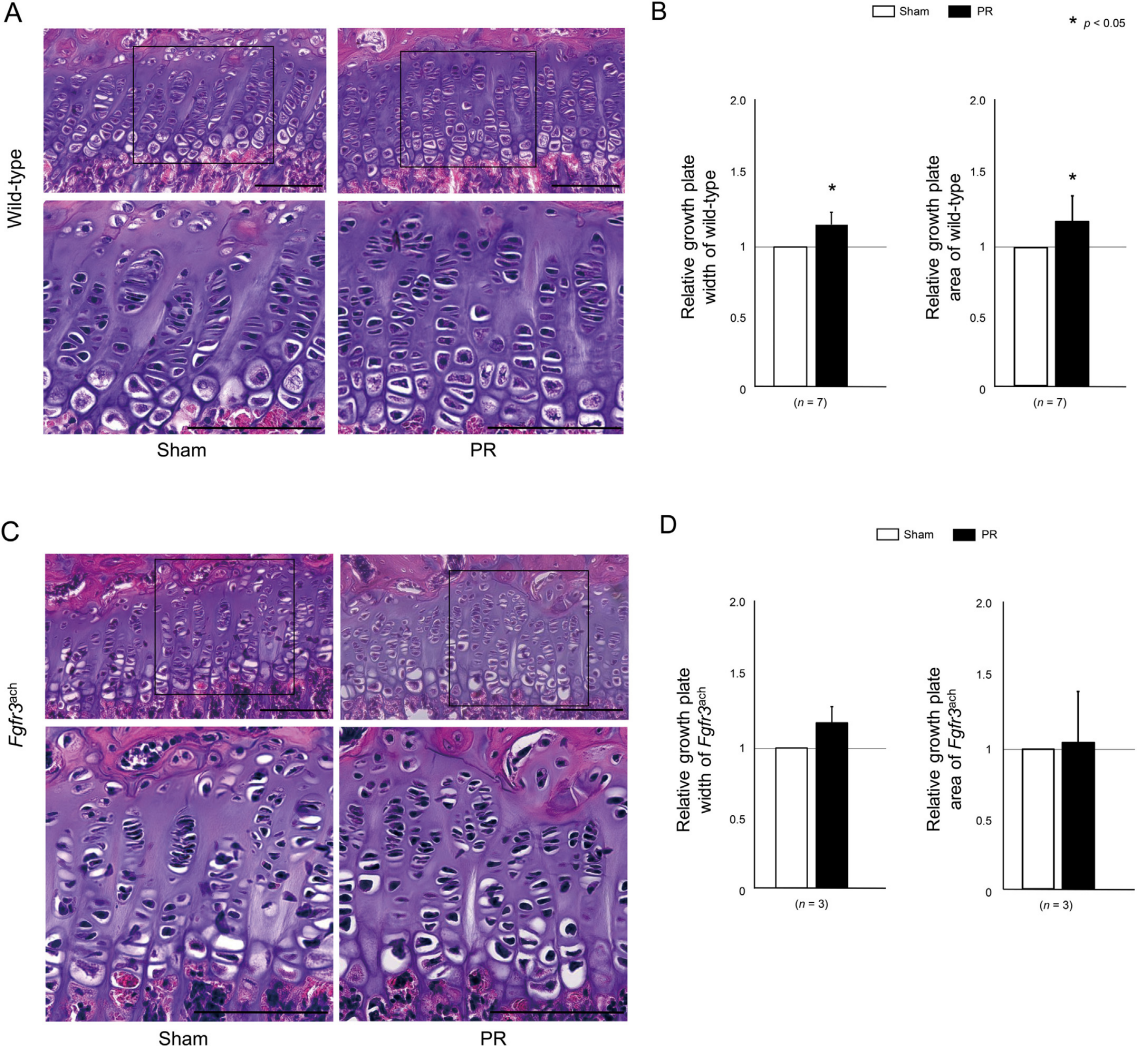
The longitudinal length of growth plate was increased after PR. (A, C) The represented histological sections show the growth plate in the proximal tibia stained with hematoxylin and eosin of wild-type (A) and *Fgfr3*^ach^ mice (C). Squared parts are magnified in each lower image. Scale bares indicate 100 μm. (B, D) Mean and SD of the longitudinal length and area of growth plate in wild-type (B) and *Fgfr3*^ach^ mice (D) are indicated. The growth plate width and area were increased after PR in wild-type and *Fgfr3*^ach^ mice, but there was no statistical difference in *Fgfr3*^ach^ mice. The statistical differences shown on each graph are analyzed by paired *t*-test.

**Fig. 6:**
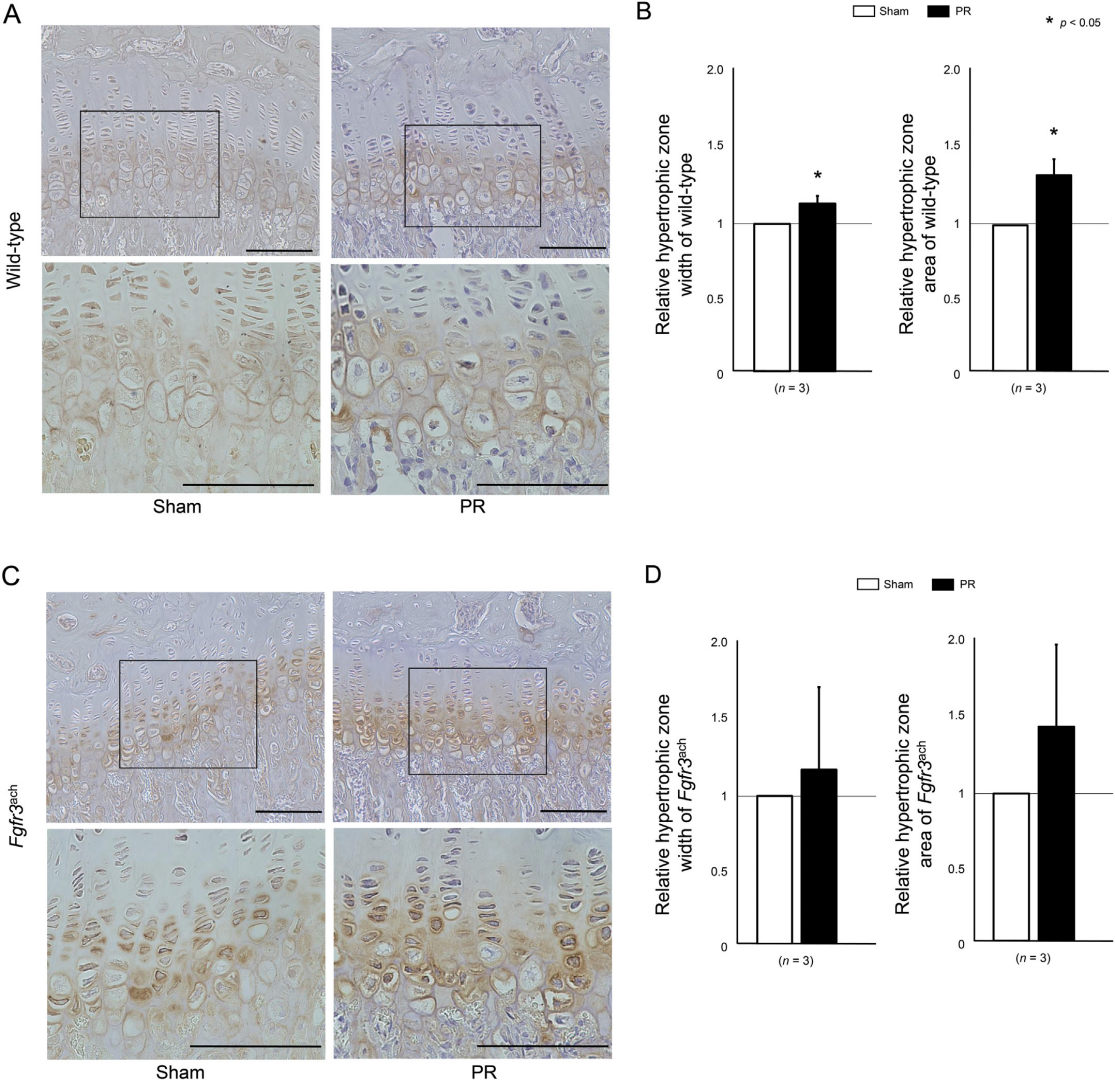
Hypertrophic zone was increased by PR. (A, C) The represented histological sections show the growth plate in the proximal tibia stained with Col X antibody of wild-type (A) and *Fgfr3*^ach^ mice (C). Squared parts are magnified in each lower image. Scale bares indicate 100 μm. (B, D) Mean and SD of each relative parameters of hypertrophic zone width and area of wild-type (A) and *Fgfr3*^ach^ mice (B) are indicated. Hypertrophic zone width and area were increased in PR of wild-type and *Fgfr3*^ach^ mice, but there was no statistical difference of these chondrocyte parameters in *Fgfr3*^ach^ mice. The statistical differences shown on each graph are analyzed by paired *t*-test.

Lastly, we evaluated the effect of PR on the morphological changes in the growth plate. The histological sections indicated that the width of the growth plate was enhanced after PR in wild-type mice (Fig. 5A). The quantitative analysis revealed that the width and area of growth plate were significantly increased after PR treatment (Fig. 5B). The similar results were observed after PR in *Fgfr3*^ach^ mice (Fig. 5C) although relative width and area of growth plate did not show significant differences (Fig. 5D). In addition, the immunohistochemical images of Col X staining indicated that PR increased hypertrophic zone width and area in wild-type mice (Fig. 6A and B), while there was no statistical difference in these parameters in *Fgfr3*^ach^ mice (Fig. 6C and D).

## 4 Discussion

This study was conducted to examine the feasibility of PR for short stature in ACH. The effect of PR on the bone growth was significant in wild-type mice but not considerable in *Fgfr3*^ach^ mice. This may be related to originally limited growth potential in mutant mice. The PR could provide more favorable acceleration of longitudinal growth when performed for the bones with vigorous growth capacity.

Although the patients with ACH could have greater gained height with fewer complications after limb lengthening surgery (Kim et al., 2014), only one clinical result of periosteal procedure was observed for ACH patients (Edwards et al., 1994). No mouse model of PR was also been reported. Experimental mouse models are beneficial because we can elucidate disease-specific effect of PR using *Fgfr3*^ach^ mice, which expresses the human achondroplasia mutation G380R. The current study could provide the effect of PR on ACH.

The increase in bone volume was more pronounced than the increase in bone length after PR treatment. This may result from increased anterior bowing of the tibia with tethering effects of posteriorly located fibula. Ali and Dickson (1994) demonstrated that tibial length was increased by 4.2% with only partial fibular excision in a rat model. They further showed tibial overgrowth in 19.7% after the combination treatment of partial fibular excision and periosteal procedure for tibia. Although simultaneous fibular excision could provide the exact measurements of tibial growth after PR, significant increase in bone volume after PR in both mice indicated some positive effects of PR on the bone growth.

Several authors demonstrated that activation of hypertrophic chondrocytes associated with an increase in size of the cells could be related to accelerated bone growth after PR. Positive linear relationship between volume of hypertrophic chondrocyte and the growth rate of longitudinal bone has been shown in an animal model (Barreto and Wilsman, 1994). Increase in number and size of hypertrophic chondrocytes after PR we showed in this study indicated an evidence of promoting bone growth rate by periosteal procedure.

Changes in bone morphometry has been reported after periosteal procedures. Some percentage of pathological fractures was demonstrated after the periosteal stripping of the lower extremities in dogs and monkeys (Sola et al., 1963). The periosteal stripping in a mouse model resulted in significantly decreased cortical thickness, cross-sectional area, bone volume, and polar moment of inertia as well as reduced peak load, stiffness, and energy to failure (Mercurio et al., 2012). In agreement with these reports, PR decreased density of metaphyseal trabecular bone in both mice models in the current study. A molecular signaling study after PR demonstrated that Indian hedgehog (IHH) was highly expressed in the hypertrophic zone of the growth plate in a lamb model (von Rechenberg et al., 2010). Activated IHH signaling promoted bone formation, but the resulting bone was very fragile and porous due to severe bone resorption (Mak et al., 2008). Thus, PR would increase growth plate width and decrease trabecular bone density via enhanced IHH signaling. On the other hand, IHH signaling in growth plate was decreased in transgenic mice with gain-of-function mutation in FGFR3 (Chen et al., 2001). Less effect of PR on promoting bone length was observed in *Fgfr3*^ach^ mice since the abnormally decreased IHH signaling would be beyond the PR.

There are several limitations in the current study. First, this is a cross-sectional study evaluating only one time point (one week after the second PR). Long-term follow-up may provide different results from this study. Second, we did not perform partial fibular excision. Third, we did not examine the effects of PR on bone growth using different mice model of ACH, which may have different bone and cartilage metabolism.

In conclusions, PR promoted longitudinal bone growth with an alteration of growth plate structure in wild-type mice. In *Fgfr3*^ach^ mice, however, the growth promotion after PR was less effective. Metaphyseal bone density was decreased in both wild-type and *Fgfr3*^ach^ mice. Periosteal procedure may have a limited effect of longitudinal bone growth in patients who have a poor growth capacity of the physis, such as ACH.

The following are the supplementary data related to this article.

## Transparency document

Transparency document Image 1

## CRediT authorship contribution statement

**Shinya Kaneko:**Methodology, Investigation, Writing - original draft, Validation.**Masaki Matsushita:**Methodology, Investigation, Formal analysis, Data curation, Writing - original draft, Validation.**Kenichi Mishima:**Formal analysis, Data curation, Validation.**Yasuhiko Takegami:**Formal analysis, Data curation, Validation.**Shiro Imagama:**Writing - review & editing, Validation.**Hiroshi Kitoh:**Methodology, Writing - review & editing, Validation.

## Declaration of competing interest

All authors state that they have no conflicts of interest.
